# Reconsiderations on the use of pipeline embolization device in the treatment of intracerebral aneurysms with special angioarchitecture: fetal PCA, AVM, V-B junction and DAVF

**DOI:** 10.1186/s41016-018-0133-8

**Published:** 2018-10-01

**Authors:** Yupeng Zhang, Peng Yan, Yuntao Di, Fei Liang, Yuxiang Zhang, Shikai Liang, Chuhan Jiang

**Affiliations:** 1 Beijing Neurosurgical Institute and Beijing Tiantan Hospital, Capital Medical University, Beijing, 100050 China; 2 Department of Neurosurgery, The People’s Hospital of Tangxian County, Tangshan, Hebei China; 3 Department of Neurosurgery, Beijing Tsinghua Changgung Hospital, Tsinghua University, Beijing, China

**Keywords:** PED, Aneurysm, AVM, Fetal PCA, V-B junction, DAVF

## Abstract

**Background:**

Pipeline embolization device (PED) has proved its safety and efficacy in the treatment of intracranial large and giant side-wall aneurysms. With the accumulation of treatment experience, it is an inevitable trend to expand its off-label use on aneurysms. Whether flow diversion is safe and efficient in cases with special angioarchitecture has rarely been explored.

**Methods:**

We performed a retrospective analysis of 210 consecutive patients treated by PED for intracerebral aneurysms in our center. Except for aneurysm, those patients also presented with special angioarchitecture: Fetal PCA, AVM, V-B junction and DAVF.

**Results:**

Nine patients were qualified for the study. 1 was with fetal PCA, the aneurysm remained patent on 4-month follow-up. 2 with ipsilateral AVMs, one patient died due to brain hemorrhage 20 days after the operation, the other one was only partially embolised on 6 month follow up. 3 aneurysms located at V-B junction, angiographic follow up on 3 months demonstrated no complete occlusion of both the aneurysms, the other patients were still on follow up. All of the 3 cases with concomitant DAVF are completely occluded during short to midterm follow up.

**Conclusions:**

PED for aneurysms incorporated the fetal PCA and V-B junction might meet a high propensity for incomplete occlusion during short term follow up. Aneurysm with ipsilateral AVM is not suitable for PED treatment due to the risk of hemorrhage and incomplete occlusion during midterm follow up. For aneurysm with concurrent DAVF, PED treatment is safe and efficient relatively in one session or by staged operation.

## Background

Since its approval by FDA in 2011, PED (ev3, USA) has proved its safety and efficacy in the treatment of large and giant intracranial aneurysms from the petrous to the superior hypophyseal segment of the internal carotid artery [1, 2]. As the experience accumulated, the off-label use has been expanded to aneurysms of other locations [3–7], small aneurysms [8], blood blister like aneurysms [9, 10], recurrent aneurysms [11–13] and ruptured aneurysms [14]. Though promising, there are also reported data related to unfavorable outcome [15], and selection of indication for treatment has gain much attention nowadays [16]. The treatment effect of PED is accredited to the flow diversion effect, and aneurysms with concomitant complex angioarchitecture might result in disturbed flow. So whether the application of PED in these settings are suitable remains to be clarified. Here, we report the outcomes of 9 patients with 10 aneurysms that incorporated at least one of the following special angioarchitecture: fetal PCA, AVM, V-B junction and DAVF.

## Methods

We retrospectively reviewed 210 consecutive patients who treated by PED between July 2015 to November 2016. Among those patients, 9 patients were identified with a special angioarchitecture that were qualified for the study, namely the aneurysm was related to fetal PCA, ipsilateral AVM, V-B junction and DAVF. The study was approved by the institutional review board of our center. We collected the clinical data and angiographic images of each patient, the clinical outcome was recorded as modified Rankin Scale (mRS) at short to midterm follow-up (Table 1). All patients were treated with dual antiplatelet therapy consisting of 100 mg of aspirin daily and 75 mg of clopidogrel for at least 5 days before the procedure. The inhibition rate was confirmed by TEG. AA% above 75% and ADP% above 50% were considered appropriate for treatment, the required MAADP was 31 mm–47 mm. Systemic heparin was used to achieve an activated clotting time of ≥250 s. We adopted routinely a triaxial system in the deployment of PED for anterior circulation aneurysm, however for aneurysms located on posterior circulation, a biaxial system consisting of 6Fr guiding catheter and Marksman microcatheter was sufficient. The concurrent AVM and DAVF are embolised via a transarterial approach by Onyx (ev3, USA).

**Table 1:** Summary of patients and aneurysms treated with pipeline

Case no.	Gender	Age (yrs)	Location & characteristics	Aneurysm status	Aneurysm Size (mm)	Adjunctive treatment	PED size (mm)	FU (days)	AN Status	mRS
1	F	61	Rt* pcomA+fetal PCA	rIAs§, SAH 12 days ago	3.0*4.0	coiling	4.5*20	111	incomplete occlusion	0
2	F	29	cavernous segment of ICA with ipsilateral AVM	UIAsII	16.0*18.0	coiling+leave the AVM untreated	3.75*30	201	incomplete occlusion	0
3	M	38	Lt† AN (OphA‡) + AVM	rIAs	5.0*6.0	leave the AVM untreated	4.75*18	20	NA	6
4	F	34	V-B + AVM	UIAs	8.5*5.0	coiling	4.25*25	94	incomplete occlusion	0
5	F	8	V-B junction	UIAs	19.9*15.4	coiling+occlusion of the contralateral VA with balloon	4.25*35	94	incomplete occlusion	0
6	F	61	V-B junction	UIAs	20.1*32.1	coiling+occlusion of the contralateral VA with coils	4.25*35 4.25*35	24	NA	NA
7	F	56	Rt AN (OphA) + transverse-sigmoid sinus	UIAs	2.9*3.9	Treat the DAVF with Chapot’s technique	4.0*16	316	complete occlusion	0
8	M	56	Rt VA+ Lt DAVF+Fenestration of VA	UIAs	14.9*6.5	Treat the DAVF with Onyx	4.5*30	318	complete occlusion	0
9	F	43	Lt AN (OphA) + DAVF	UIAs, multiple, small	2.5*2.8 (1) 2.5* 3.1(2)	Treat the DAVF with Onyx	3.75*20	12	complete occlusion	0

*Rt = for right

† Lt = for left

‡ OphA = Ophthalmic Artery

§ rIAs = ruptured intracerebral aneurysms

II UIAs = unruptured intracerebral aneurysms

## Results

We identified 10 aneurysms from 9 patients that met our inclusion criteria (Table 1). Our cohort included 1 patient with fetal PCA, 2 patients with ipsilateral AVM, 3 patients located on V-B junction and 3 patients with concomitant DAVF. All of the patients except Case NO. 3 were scored 6 according to the mRS system and were free from any neurological deficit. Case NO.3 died 20 days after operation in local hospital due to brain hemorrhage.

The size of aneurysms ranged from 2.5 mm*3.1 mm to 20.1 mm*32.1 mm. Case NO. 6 was treated one month ago and has not been followed up with DSA. The other two aneurysms at V-B junction as well as the aneurysms with fetal PCA or ipsilateral AVM remained canalized (Raymond Scale Score 3) on short to midterm angiographic follow up, even though the aneurysms decreased in size to some extent. On the contrary, all of the 4 aneurysms with concurrent DAVF achieved complete occlusion either through staged procedure or within one single session.

We discontinued clopidogrel use for Case No. 4 and 5, and the dual antiplatelet therapy for Case NO. 1 and 2, hoping that this modification of drugs would accelerate the occlusion of the aneurysm. None of these 4 patients received further invasive treatment and was under further angiographic follow up.

### Illustrative cases

#### Fetal PCA aneurysms (case 1)

**Fig. 1:**
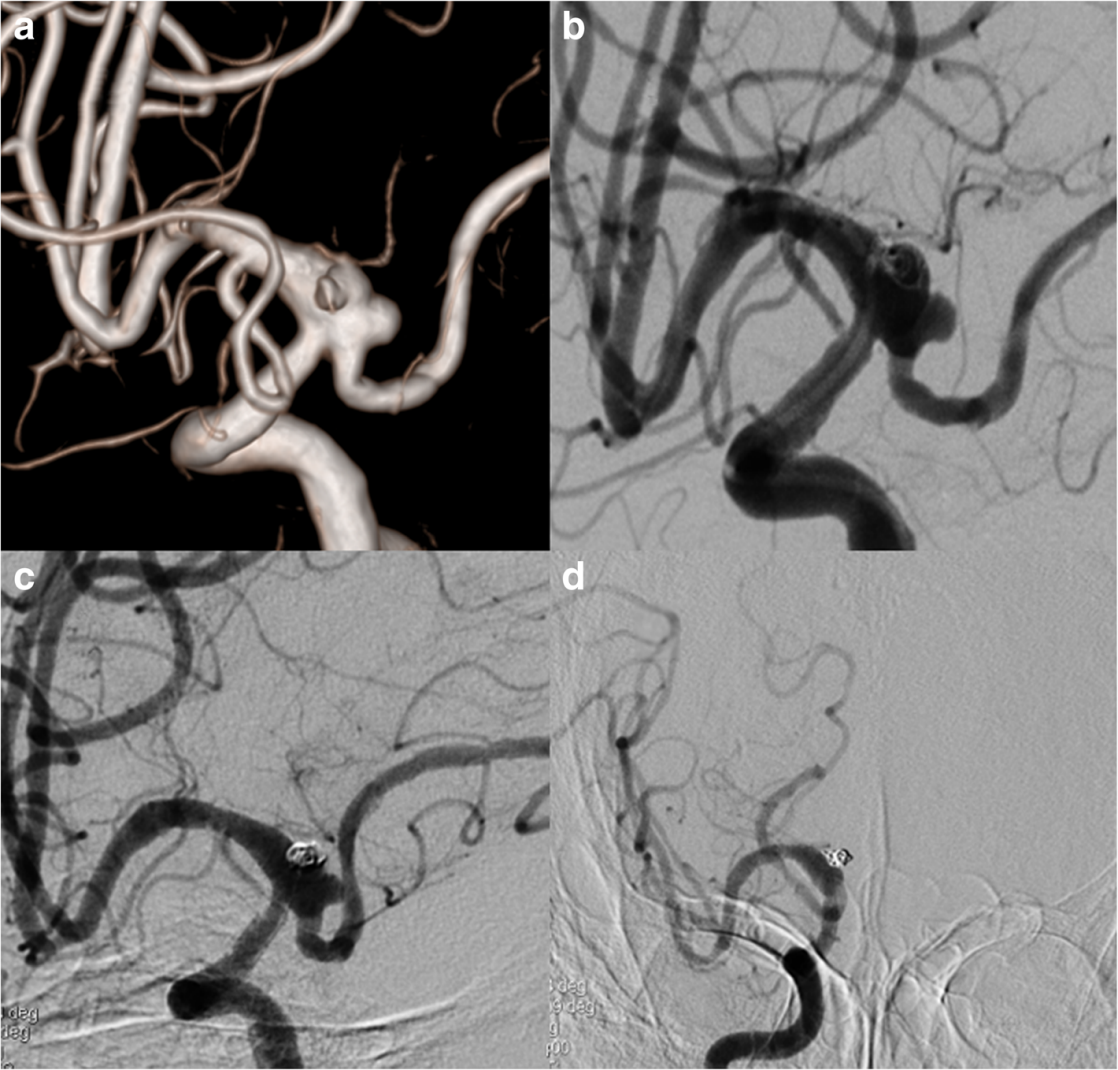
3D DSA image of Case NO. 1 before the treatment (**a**), immediate postoperative angiogram (**b**) and on follow-up (**c**), note that the right A1 was not patent, the distal part of right ACA was supplied by compensatory flow from the AComA (**d**)

This was a 61 years old female presented with a ruptured PCoA aneurysm which incorporated the origin of a right fetal PCA. The aneurysm was measured 3.0 mm*4.0 mm with a relative wide neck and two daughter aneurysms (Fig. 1a). We postponed the procedure 20 days after the onset of SAH to avoid the potential risks of dual antiplatelet therapy. The daughter aneurysm located superiorly was considered to be the rupture point, so we first loosely embolised it with three coils measured 2 mm*3 cm, 1.5 mm*2 cm, 2 mm*4 cm, then we deployed a 4.5 mm*20 mm PED from M1 segment to supraclinoidal segment of ICA covering the neck of the aneurysm (Fig. 1b**)**. No further coiling of the aneurysm was performed in consideration of the future patency of fetal PCA. The follow up angiogram on 4 months demonstrated the patency of the fetal PCA and the aneurysm has slightly decreased in size (Fig. 1c), however there remained contrast agent filling in the aneurysm sac. Note that the right A1 is no longer patent and the patient was asymptomatic due to the compensatory flow from the AComA (Fig. 1d). So we discontinued the dual antiplatelet therapy and leave this patient on MRI follow up, we will not retreat the patient unless the patient will present with headache and enlargement of the aneurysm on MRI image.

#### Aneurysm with ipsilateral AVM (case 2, case 3)

**Fig. 2:**
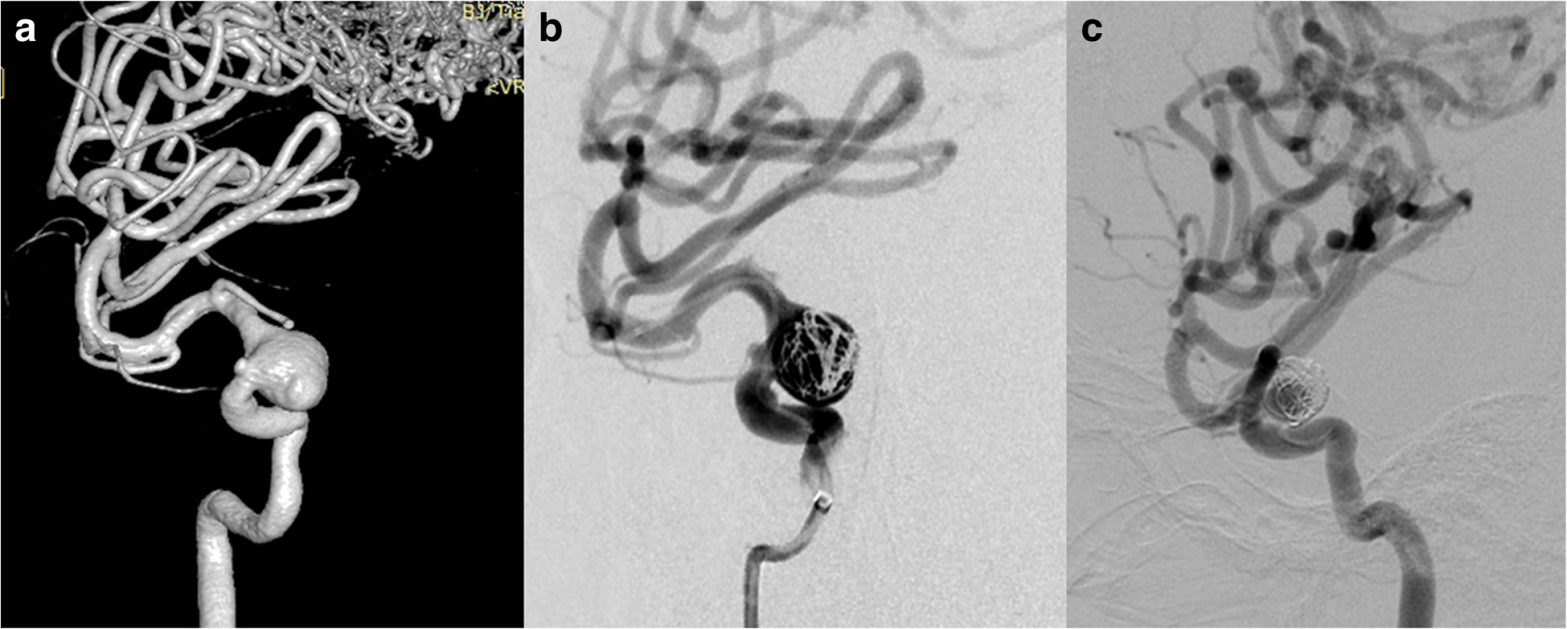
Case NO. 2 was a young female with a large cavernous aneurysm and a coexisting Spetzler-Martin grade 3 AVM of the right temporoparietal region (**a**). Immediate postoperative angiogram (**b**) and 7 months angiographic follow up (**c**) indicated that the size of the aneurysm has decreased but still partial embolised

**Fig. 3:**
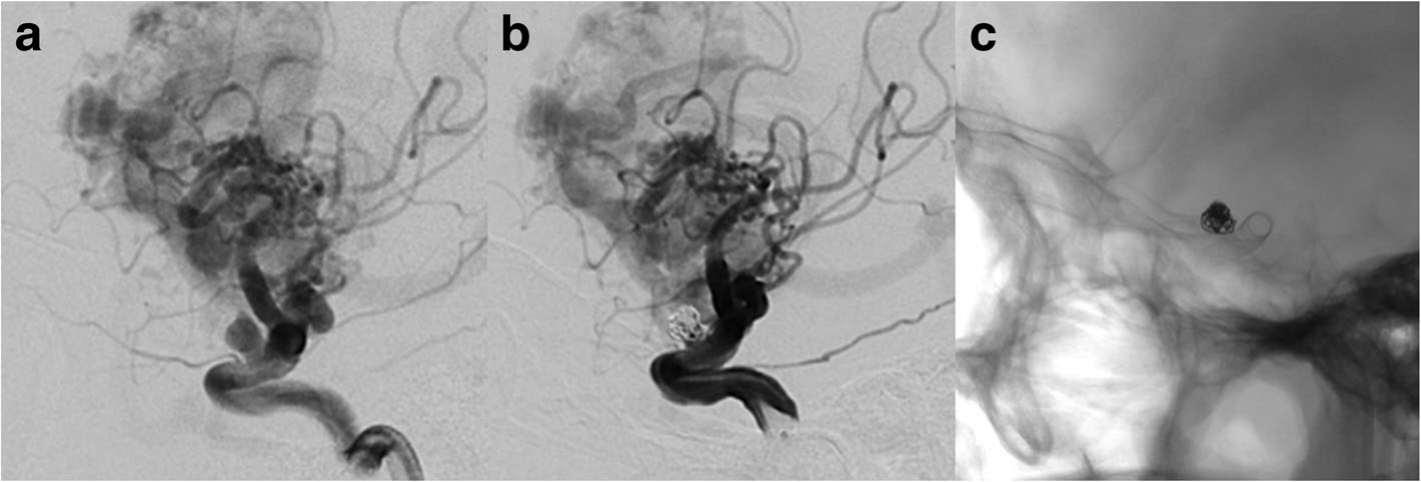
Case NO. 3 was an ophthalmic aneurysm with an AVM (Spetzler-Martin 3) (**a**), we placed one PED (4.75 mm*18 mm) and further occluded it with 8 coils (**b**, **c**)

We have treated 3 patients (case 2, case 3, case 6) in total with an aneurysm whose parent artery was also the feeder of an ipsilateral AVM. Case 2 was a young female with a Spetzler-Martin grade 3 AVM of the right temporoparietal region. This diagnosis was confirmed 14 years ago when epilepsy firstly onset. This patient was on regular image follow up, and the DSA performed 10 months ago revealed a newly formed cavernous aneurysm (Fig. 2a). Since the aneurysm is the vulnerable part for rupture, we deployed a PED of 3.75 mm*30 mm to cover the neck, then loosely coiled the dome of the aneurysm with 3 coils, namely, Presidio 10 coil 7 mm*30 cm (Codman, USA), MicroPlex 10 Helical 10 mm*30 cm and 9 mm*30 cm (Stryker, USA). The total packing volume was 2.08% (Fig. 2b). 7 months angiographic follow up (Fig. 2c) demonstrated that the size of the aneurysm has decreased but still partial embolised. Case No. 3 was a 38-year-old male admitted into our department with an AVM (Spetzler-Martin grade 3) and an ophthalmic aneurysm which ruptured 45 days ago (Fig. 3a). The aneurysm measured 5 mm in length and 6 mm in width and there existed a daughter sac which presumed to be the rupture point. We then placed one PED (4.75 mm*18 mm) and further occluded it with 8 coils (Fig. 3b, c). The packing volume reached 40%. The whole procedure completed without any technical difficulties and the patient discharged without any neurological deficits, however, the patient died 20 days thereafter due to brain hemorrhage in local hospital.

#### Aneurysm located on V-B junction (case 4, case 5, case 6)

**Fig. 4:**
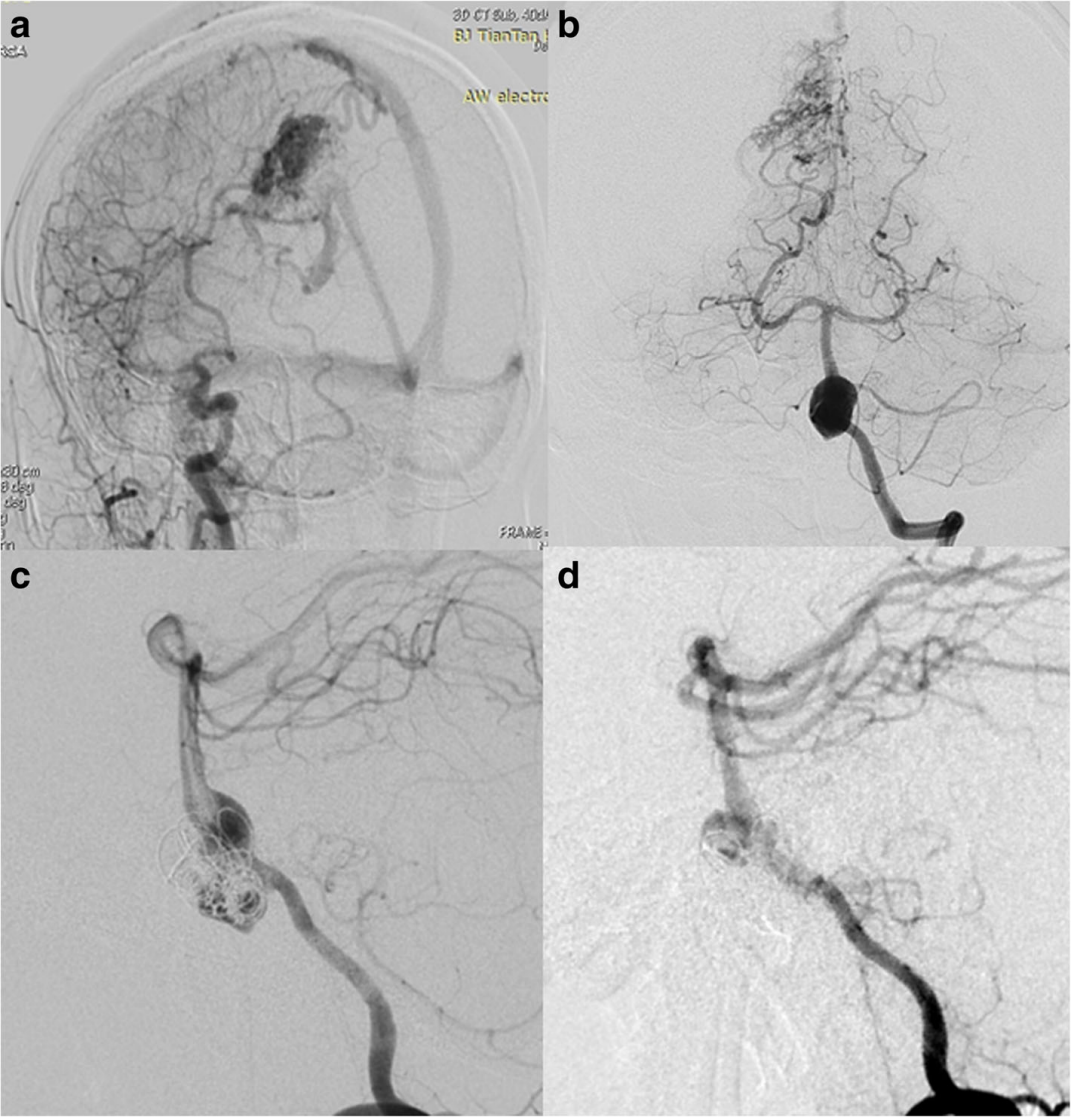
Case No. 4 has a V-B junction aneurysm with coexisting AVM supplied by both anterior (**a**) and posterior circulation (**b**). We deployed the PED from the lower 1/3 of basilar artery to the left VA and further coiled the aneurysm loosely (**c**). 3 months follow up indicated incomplete occlusion of the aneurysm (**d**)

**Fig. 5:**
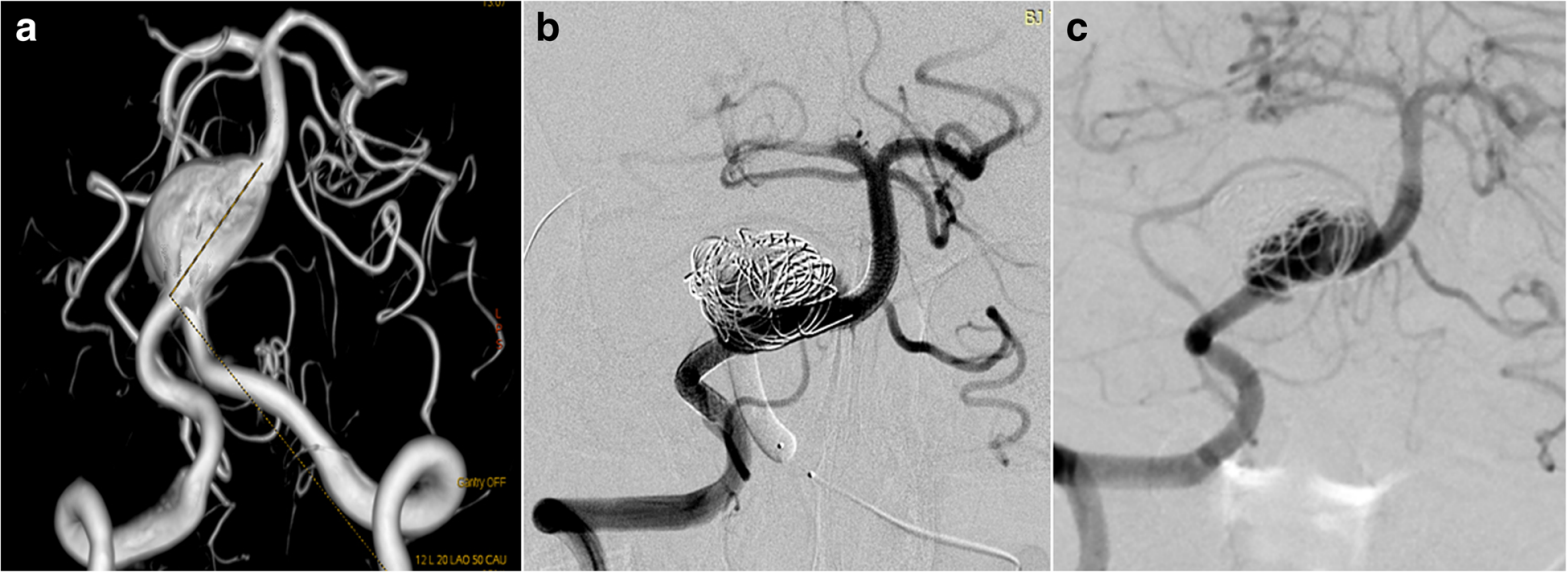
Case No. 5 was an 8-year-old girl with a large V-B junction dissecting aneurysm (**a**), we overlapped two PEDs, coiled the aneurysm and sacrificed the left VA with a detachable balloon (**b**). At 3-month follow-up, the aneurysm was still patent but decreased in size (**c**)

**Fig. 6:**
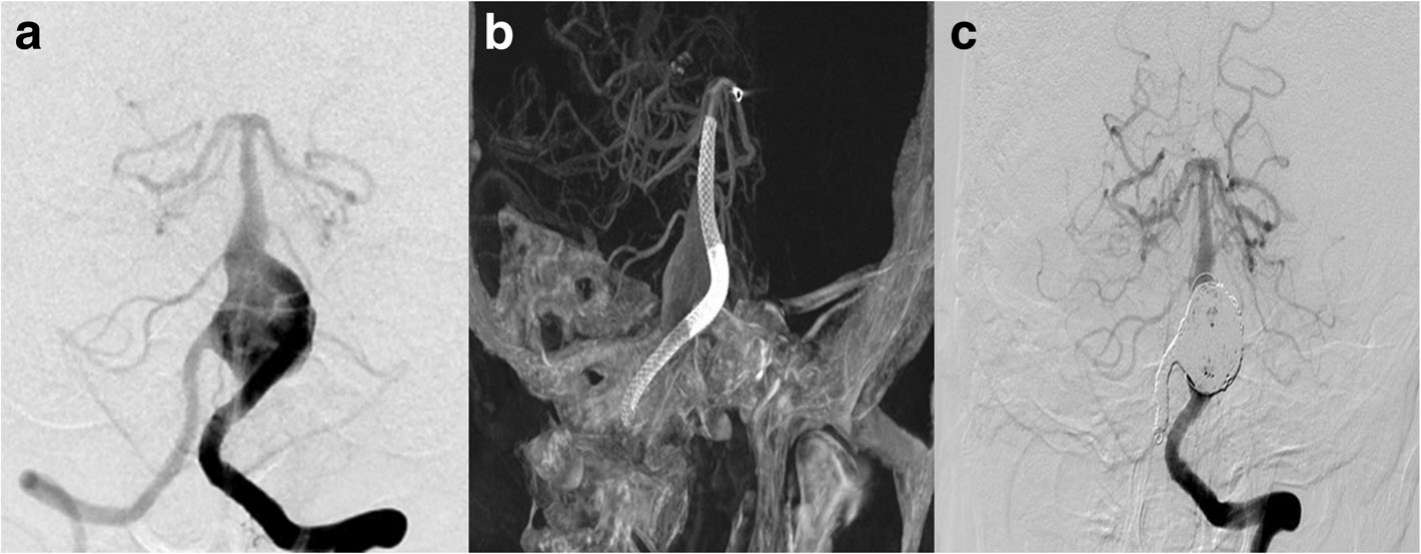
Case No. 6, a V-B junction aneurysm (**a**) treated by overlapping 2 longest PEDs (4.25 mm*35 mm) (**b**) to enhance the flow diversion effect and further occluded the contralateral VA with coils (**c**). Note that the distal PED covered the mid-basilar perforators

Case No. 4 was 34-year old female complained a gradually aggravated headache for 3 months. DSA showed a V-B junction aneurysm. Besides the aneurysm, the patient suffered from an AVM (Spetzler-Martin 3) that received feeding arteries both from the anterior posterior circulation (Fig. 4a, b). We deployed the PED (4.25 mm*25 mm) from the lower 1/3 of basilar artery to the left VA and further coiled the aneurysm loosely to achieve favorable stasis of flow (Fig. 4c). 3 months follow up indicated incomplete occlusion of the aneurysm (Fig. 4d). Case No. 5 was an 8-year-old girl presented with ataxia, vomiting and nausea. DSA indicated a large V-B junction dissecting aneurysm (Fig. 5a). Learned from the experience of case 4, we decided to telescoping two PEDs (4.25 mm*35 mm) to achieve a better flow diversion effect. Control angiography suggested that there was no apparent stasis of angiographic agent due to the persistent blood flow from the contralateral VA. So we decided to coil the aneurysm and concomitantly to occlude the aneurysm with a detachable balloon (Balt, France) (Fig. 5b). The patient recovered from general anesthesia smoothly and discharged without any neurological deficit. 3 months follow up angiography indicated a certain degree of decrease in size and the patient was free from any symptom, yet the aneurysm was still partially embolised (Fig. 5c). So, knowing that the V-B junction aneurysm is quite refractory in nature, we treated case No. 6 (Fig. 6a) By overlapping 2 longest PEDs (4.25 mm*35 mm) to enhance the flow diversion effect and further occluded the contralateral VA with coils (Fig. 6b, c). It is inevitable to cover the perforating branches of basilar artery if you chose a 35 mm long PED. The patient experienced transient visual loss of both eyes, however, the patient completely recovered from these symptoms one day after operation. This patient is still on follow-up.

#### Aneurysm with concomitant DAVF (case 7, case 8, case 9)

**Fig. 7:**
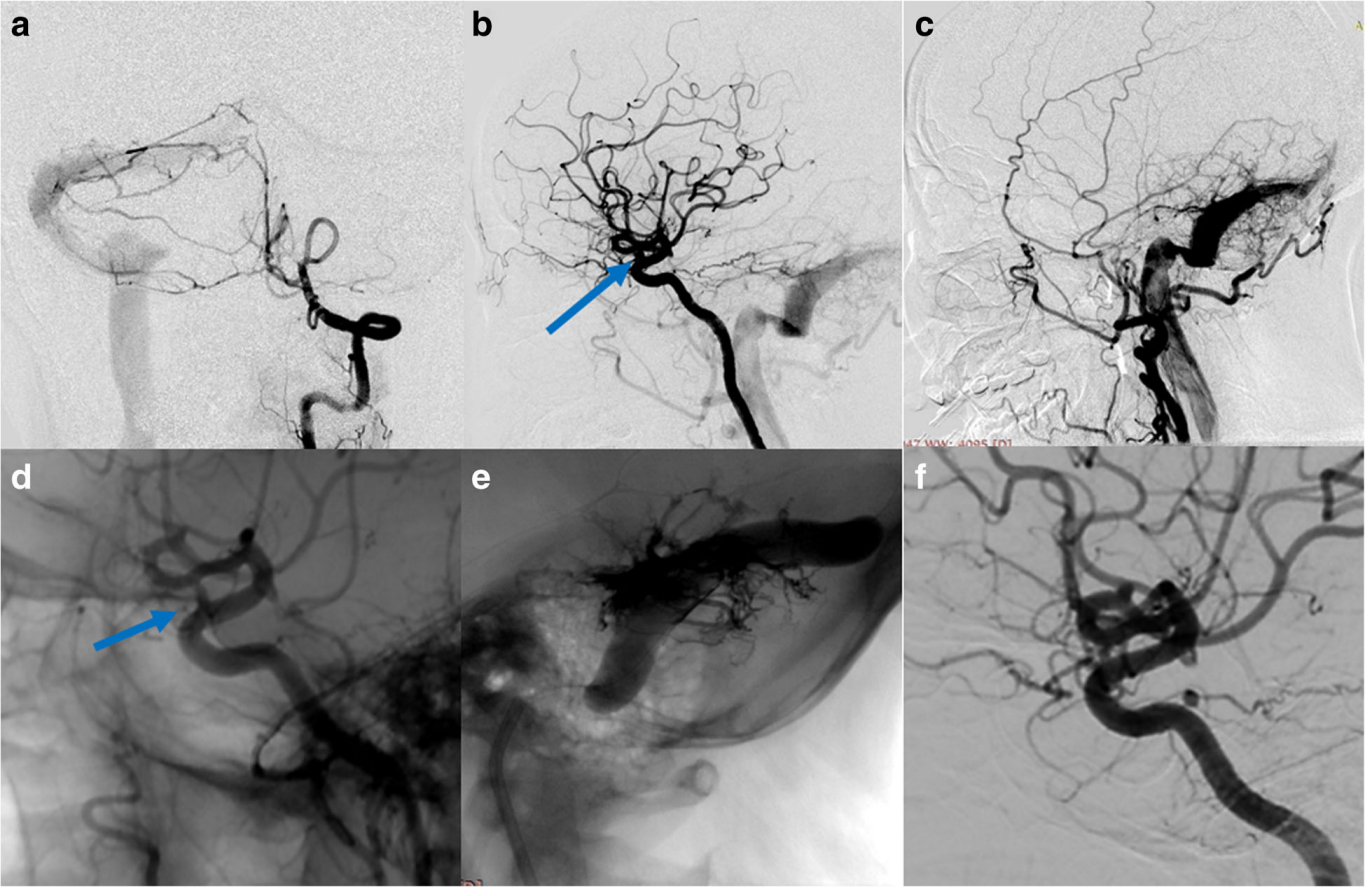
Case No. 7 was a 56-year-old female with a transverse sinus DAVF whose feeding arteries were from right VA (**a**), right petrosquamous branch of ICA (**b**), right middle meningeal artery and right occipital artery (**c**). The aneurysm on ophthalmic segment of ICA (**b**, blue arrow) was treated by a PED without further coiling and was incompletely occluded (**d**, blue arrow). The fistula was embolised using the Chapot’s technique (**e**). The 6 month follow up demonstrated complete occlusion of the aneurysm (**f**)

We treated 3 cases of aneurysm with concomitant DAVF either in one single session (Case No.7) or by a staged procedure (Case No. 8, Case No.9). Case No. 7 was a 56-year-old female with a transverse sinus DAVF whose feeding arteries were from right VA (Fig. 7a), right petrosquamous branch of ICA (Fig. 7b), right middle meningeal artery and right occipital artery (Fig. 7c). The aneurysm measured 2.9 mm*3.9 mm and was located near the origin of the right ophthalmic artery. We deployed a PED (4.0 mm*16 mm) to treat the aneurysm without further coiling it (Fig. 7d). We applied the Chapot’s technique to protect the sinus during embolization of the DAVF through the right middle meningeal artery (Fig. 7e), then we deflated the Copernic balloon (Balt, France) and embolised the residual part of fistula through the right occipital artery. The 6 month follow up demonstrated complete occlusion of the aneurysm (Fig. 7f) and minimal residual of DAVF. We performed staged procedures for Case NO.8 and NO.9, we first treated the anterior fossa DAVF (Case NO.8) and transversus sinus DAVF (Case NO. 9) with Onyx-18 (ev-3, USA). Three months later, when complete occlusion of the fistula was confirmed, we performed PED for both patients. For the aneurysm of Case 9, its patent artery gave branch to feed the fistula, namely, the ipsilateral petrosquamous branch. While for case NO. 8, the right vertebral artery aneurysm was not correlated with any of the feeders of the anterior fossa DAVF. None of the 3 patients experienced any neurological complications, the aneurysms all completely occluded within short-term angiographic follow-up.

## Discussion

In our series, we reported the outcomes of 9 patients with 10 aneurysms that incorporated at least one of the following special angioarchitecture: fetal PCA, AVM, V-B junction and DAVF. Except that cases with DAVF were all recovered better and the aneurysms achieved complete occlusion on short to midterm follow-up, we encounter failure to treat aneurysms with fetal PCA, AVM and located at V-B junction. This result suggested that we should limit our use of PEDs in those settings.

With reference to other reports and as manifested in our case, there were 6 patients with fetal PCA aneurysm treated with PED in literature. All of the reported cases were performed in a way that the PED was placed from the M1 segment to the ICA, expecting that the PED would divert the flow from the fetal PCA. However, none of the aneurysms occluded completely on an angiographic follow up that ranged from 3 months to 30 months, even after the discontinuation of the dual antiplatelet therapy. Peter Kan et al. [15] presumed that failure of these cases is due to the fact that fetal PCA has constant anterograde flow without competitive flow through the ipsilateral P1 segment. Flow through the fetal PCA and the aneurysm sac remains high even after deployment of PED owing to the high physiologic demand of the posterior circulation. This high flow would undermine the stasis of blood in the aneurysm. So it is reasonable to manage this kind of aneurysms either by microsurgical clipping or coiling when feasible.

In patients harboring an AVM, concurrent arterial aneurysms occur in nearly 2.7–58% patients, with 10%–20% being most often cited in the largest case series. [17]. These flow associated aneurysms are the weak points that significantly increase the risk of hemorrhage, so the treatment of these two lesions entails a thorough understanding of the hemodynamics and angioarchitecture [17–20]. Considering the complex flow pattern, deployment of a flow diverter to the aneurysm might result in unfavorable outcome. Shakur et al. as well as other authors had reported that after PED placement, the hemodynamics in distal arteries will be disrupted and contribute to delayed ipsilateral intraparenchymal hemorrhage [21, 22]. In our Case NO. 3, we are still not sure about whether the lethal hemorrhage was due to rerupture of the aneurysm or the AVM, since the patient died in a local hospital. But this case is adequate to alert our use of PED in these settings. Not only prone to rupture, the coexisting AVM might delay the occlusion of the aneurysm. Even though implanting a PED would lower the flow volume rate of the parent artery [21], the flow through the parent artery which ultimately feed the AVM remains high, this will undermine the stasis of blood in the aneurysm. However, this is only a hypothesis that requires further validation of flow dynamic data.

Reports regarding the application of PED on aneurysms at V-B junction is still rare [23–25], since covering the mid-basilar perforators predisposes the patient for brainstem stroke. By far, Munich et al. reported the largest cohort of patients with a vertebrobasilar fusiform aneurysm. At an average follow-up of 11 months, complete aneurysm occlusion was seen in 90% of the patients and 3 of the 11 patients (27%) suffered new neurological deficits postoperatively and one of these patients died [24]. Natarajan et al. has reported another series of 6 patients [23]. At an average follow up of 9 months, 5 of the 6 patients achieved complete obliteration of the aneurysm (83.3%). Only one patient in his series required a second procedure (16.7%), since DSA follow-up on 7 months demonstrated patency of the aneurysm sac. Besides that, one of the six patients experienced brainstem perforator stroke, the symptoms persisted and the mRS was 4 on 12 months’ follow-up. Despite the promising results in the literature, 1 of the 3 patients experienced a transient ischemic stroke, and DSA follow up at 3 months for the other 2 patients indicated partial embolization of the aneurysms. Two reasons might account for this disparity of results, namely the number of PEDs used and the length of follow up time. In Munich’s series, the average number of PEDs were 3.6, along with 2 Enterprise stents (Codman, USA). In Natarajan’s series, half of the patients were treated with more than 2 PEDs. Presumably, overlapping multiple PEDs will enhance the flow diversion effect, however at a higher risk of elevated ischemia rate. We only deployed one single PED for Case No. 4, the lack of adequate flow diversion might be the explanation for partial embolization. However, even though we overlapped two PEDs and sacrificed the left VA for Case NO. 5 with a balloon, the stasis was still unsatisfactory, and follow up at 3 months indicated patency of the aneurysm despite that the aneurysm indeed shrinked in size. For this case, the relatively short follow up time might be the excuse. Since the patient was free from the symptoms aforementioned, we discontinued clopidogrel and put this patient on further DSA follow up. In our experience, we take special cautions in the treatment of V-B junction aneurysms with PED, in that it requires a thorough analysis between a relative long interval for complete occlusion and the risk of overlapping multiple PEDs. Endovascular treatment of aneurysms located at vertebrobasilar junction was a demanding task. Traditional vascular approaches had unsatisfactory treatment outcome regarding the complete occlusion rate and complication rate. Flow diversion offers us with an alternative due to its capability of endoluminal reconstruction and avoids robust packing of coils in the aneurysms sac. Considering the low porosity of pipeline, we treated these aneurysms with great caution. Flow diversion treatment was initiated only when the aneurysm become enlarged or symptomatic on follow up. If left untreated at this timing, due to the increased risk of periprocedural compression of brain stem, the aneurysm will progress to a bigger volume that exclude the possibility of coiling, stent assisted coiling and flow diversion.

## Limitations

This is a retrospective review and only included a small number of patients. There exists inherent bias with such a study design. Besides that, we did not obtain hemodynamic data in patients, so we cannot illustrate how the coexistence of these angioarchitectures influenced the occlusion of the aneurysm. At last, the follow-up period is relatively short in patients with aneurysms at V-B junction, so we cannot give concrete data that our treatment modality is successful in the long term. However, our study did give us some hints on the selection of patients that will benefit from a pipeline embolization device.

## Conclusion

PED for aneurysms incorporated the fetal PCA and V-B junction might meet a high propensity for incomplete occlusion during short term follow up. The treatment of V-B junction aneurysms entails the sacrifice of the contralateral VA and overlapping multiple PEDs. We should pay special attentions to these aneurysms and seek a balance point between complete occlusion rate and the risk of ischemic stroke. Aneurysm with ipsilateral AVM is not suitable for PED treatment due to the risk of hemorrhage and incomplete occlusion during midterm follow up. For aneurysm with concurrent DAVF, the aneurysm might be safely and efficiently treated either in one session or by staged operation.

## Abbreviations

AVM: Arteriovenous malformation
DAVF: Dural arteriovenous fistula
DSA: Digital subtracted angiography
ICA: Internal carotid artery
PCA: Posterior cerebral artery
PED: Pipeline embolization device
VA: Vertebral artery
V-B junction: Vertebral-basilar junction
